# Intercostal Artery Laceration: Rare Complication of Thoracentesis and Role of Ultrasound in Early Detection

**DOI:** 10.1155/2017/6491083

**Published:** 2017-08-02

**Authors:** Wissam Mansour, Ghassan Samaha, Sandy El Bitar, Ziad Esper, Rabih Maroun

**Affiliations:** ^1^Department of Internal Medicine, Northwell Health Staten Island University Hospital, 475 Seaview Avenue, Staten Island, NY 10305, USA; ^2^Department of Pulmonology and Critical Care Medicine, Northwell Health Staten Island University Hospital, 475 Seaview Avenue, Staten Island, NY 10305, USA

## Abstract

Hemothorax is a rare but potentially fatal postthoracentesis complication. Early clinical signs may be nonspecific resulting in diagnostic delay. A high index of suspicion is vital for early diagnosis and intervention to avoid further bleeding. Following procedure, early bedside ultrasound findings can be vital for early detection. We report a case of massive hemothorax in a 63-year-old male following therapeutic thoracentesis. Diagnosis was made following highly suggestive sonographic findings prompting thoracotomy and lacerated intercostal artery cauterization.

## 1. Introduction

Hemothorax is a fatal but fortunately rare occurrence following thoracentesis. It usually results from laceration of the intercostal artery. A high index of suspicion after intervention is crucial for early detection and subsequent treatment to achieve hemostasis. Ultrasound findings of early loculated fluid reaccumulation should prompt further investigation and intervention. We will present a case of hemothorax after thoracentesis in a high risk patient and discuss the role of ultrasound in diagnosis.

## 2. Case Presentation

**Figure 1:**
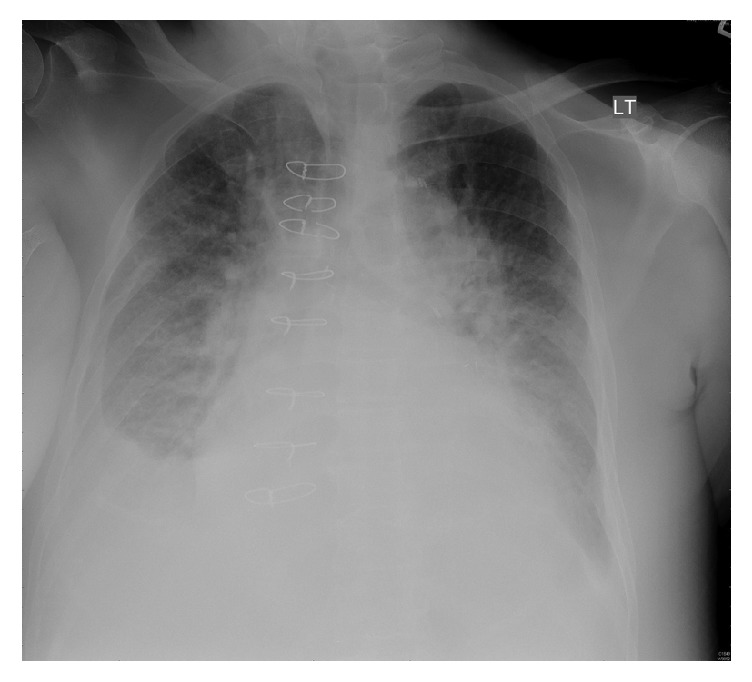
Portable chest radiograph on presentation showing bilateral basilar opacities with pleural effusions and hilar congestion.

**Figure 2:**
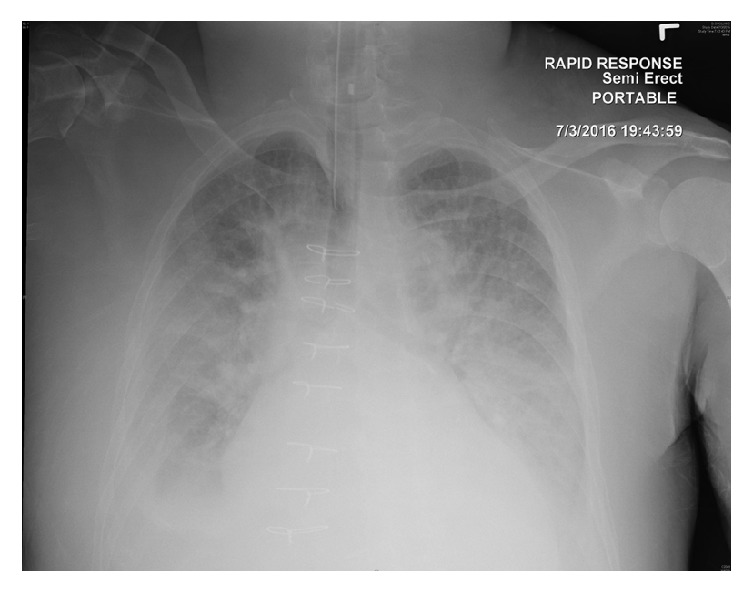
Portable chest radiograph on day 2 following intubation for respiratory distress showing worsening bilateral opacities and hilar congestion.

**Figure 3:**
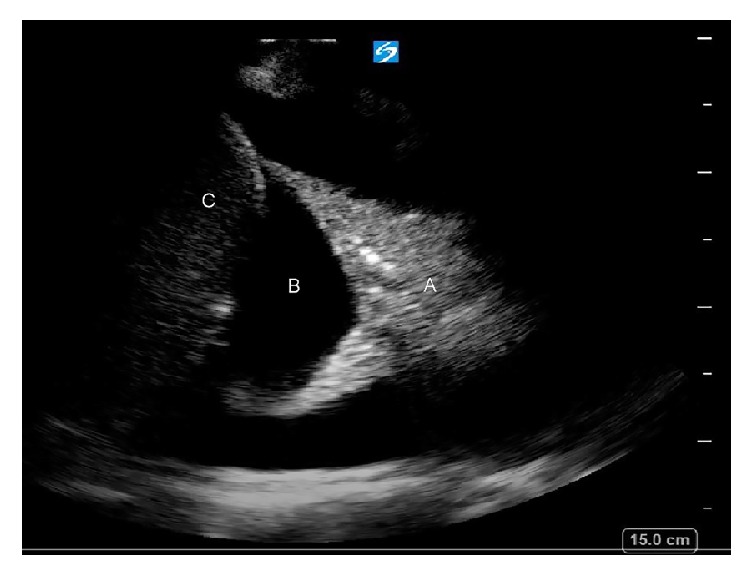
Chest US prior to thoracentesis demonstrating anechoic pleural fluid (B). A = collapsed lung parenchyma; C = liver.

**Figure 4:**
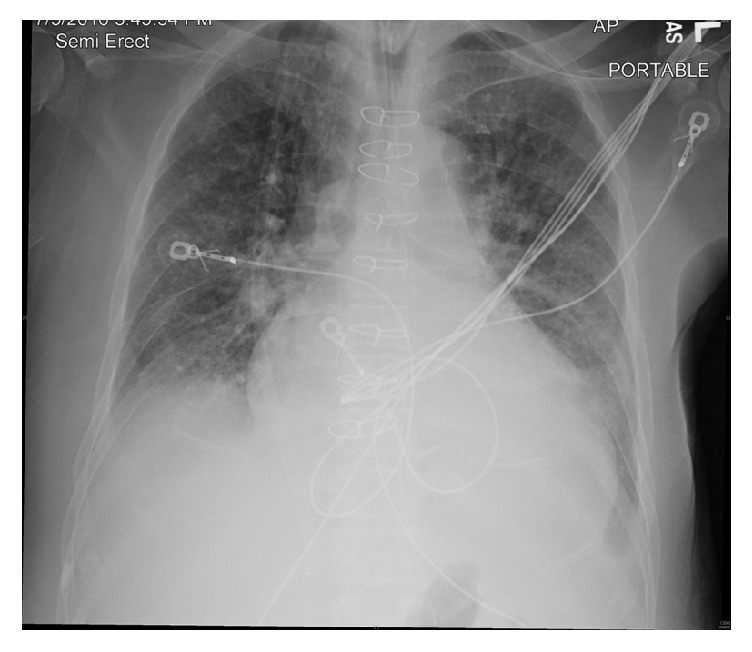
Portable chest radiograph following right-sided thoracentesis showing significant reduction in pleural effusion.

**Figure 5:**
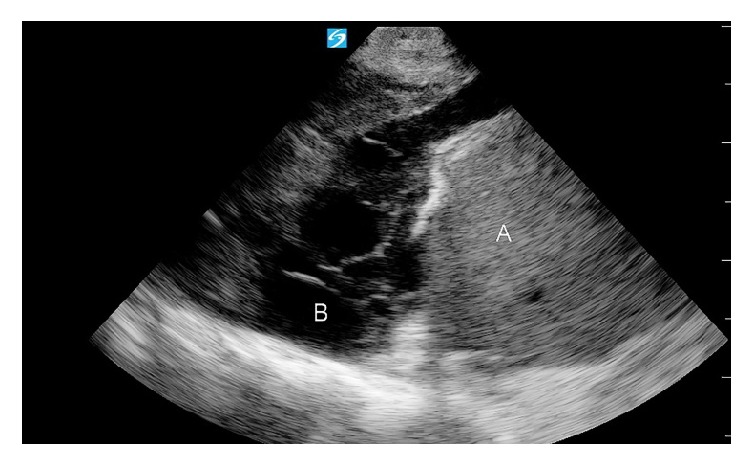
Chest US approximately 8 hours after thoracentesis demonstrating complex pleural fluid with septations and debris (B). A = liver.

A 63-year-old male presented with shortness of breath of 2-week duration. Medical history was notable for coronary artery disease status postcoronary artery bypass graft, end stage renal disease on hemodialysis, and oxygen-dependent COPD. Home medications included low dose aspirin, metoprolol, atorvastatin, hydralazine, sevelamer, and tiotropium inhaler. Upon admission patient was afebrile, with blood pressure of 135/80 mmHg, heart rate of 75 beats per minute, and oxygen saturation of 93% on supplemental oxygen 3 L/min via nasal cannula. Physical exam revealed decreased air entry over lung bases associated with bilateral lower-extremity 3+ edema. Pertinent laboratory findings included hemoglobin of 9.5 g/L, platelet count of 180 × 10^9^/L, blood urea nitrogen of 57 mg/dL, serum creatinine of 4.5 mg/dL, and INR of 1.2. A chest radiograph demonstrated bilateral pleural effusions (Figure 1). Patient underwent urgent hemodialysis for fluid overload, slightly improving his dyspnea. Hospital course was complicated on day 2 with worsening respiratory distress and hypoxemia requiring mechanical ventilation. Repeat chest radiography showed worsening bilateral lung opacifications and effusions (Figure 2). Patient was found to have low-grade fever (101.2 F) and blood work demonstrated mild elevation in WBC (13,000/mm^3^). Decision was made to perform a diagnostic/therapeutic thoracentesis to evaluate for parapneumonic effusion. Other than suspected platelet dysfunction secondary to uremia and low dose aspirin, our patient had no evident coagulopathy. He was placed in supine position with chest elevated at 45°. Bedside ultrasound revealed bilateral anechoic large effusions (Figure 3). Once the fluid was located over the right midaxillary line, skin was prepped and draped. Lidocaine 1% was used for local anesthesia. An 8-French catheter was inserted over the tract of the superior aspect of the 9th rib and 1,100 mL of yellow fluid was obtained. The patient tolerated the procedure well. Chest radiograph obtained immediately after thoracentesis revealed decreased right-sided effusion (Figure 4). Fluid analysis suggested a transudative effusion with an erythrocyte count of 109/mm^3^. Patient's oxygenation improved and he was successfully extubated. Overnight, approximately 8 hours after thoracentesis, the patient developed shortness of breath. Vital signs revealed a maintained blood pressure at 100/65 mmHg, a heart rate of 100 beats per minute, and an oxygen saturation of 88% on 3 L/min supplemental oxygen via nasal cannula. Bedside lung ultrasound demonstrated large bilateral pleural effusions with septations and debris on the right (Figure 5). Hemothorax was suspected and a Pigtail catheter was placed draining 2 L of bloody fluid. Repeat blood work revealed drop of hemoglobin to 7.4 g/L from a baseline of around 9 g/L. Patient received desmopressin injection and one unit of packed red blood cell (PRBC) transfusion. Urgent right lateral thoracotomy was performed revealing a bleeding posterior intercostal artery (ICA) in the lateral chest wall at the level of the 8th intercostal space. The culprit vessel was cauterized and clipped. Intraoperatively the patient received two units of PRBC with estimated blood loss of 1.5 L. Patient had an uncomplicated postoperative course and was extubated on the next day. Hospitalization was however further complicated with non-ST elevation myocardial infarction and death on hospital day 8.

## 3. Discussion

Thoracentesis is a common bedside procedure performed to evaluate pleural effusions. It carries a diagnostic and therapeutic value. The procedure is indicated in most cases to determine the nature of the fluid and for potential identification of the underlying etiology. In general it is a well-tolerated intervention with low incidence of complications [1]. Bleeding events following the procedure range from local puncture site bleeding to hemothoraces. The latter have an incidence of 0.01–0.1% [2]. Underlying pathophysiology involves laceration of the posterior ICA. The degree at which coagulopathy can be considered a risk factor for hemothorax remains an issue of controversy. Despite sparse data, caution is advised in patients with advanced renal insufficiency and severe coagulopathy [2]. Consensus guidelines, including those from the Interventional Radiological Society of Europe and the British Thoracic Society, recommend avoiding routine thoracentesis in cases of prolonged INR more than 1.5–2, and platelets less than 50,000/*μ*l. Clopidogrel should preferably be held for 5 days prior to procedure, whereas aspirin can be continued with no significant increase in bleeding risk [3, 4].

While it is common practice to advance the thoracentesis needle above the superior aspect of the rib to help avoid the vascular bundle, this might not apply to all cases. The ICA typically runs along the subcostal groove; however, studies demonstrated that the degree to which the ICA is exposed within the intercostal space varies significantly [5]. ICA exposure increases with advanced age, in patients with previous sternotomy, and in caudal intercostal spaces [5–7]. Dewhurst et al. concluded ICA tortuosity to be minimal lateral to the angle of the rib, an area close to midaxillary line [5]. Based on this predicted ICA course, a lateral approach is recommended for pleural procedures through the triangle of safety [2]. Despite adhering to safety precautions, the risk of ICA laceration persists mostly due to anatomical variations. Recent reports suggest the use of vascular ultrasound with color flow to evaluate for these variations prior to thoracentesis [8, 9]. Compared to CT angiography, vascular ultrasound demonstrated a high sensitivity reaching 90% in detection of vulnerable exposed ICA [8]. The utilization of this technique in high risk patients is becoming more popular; however, its impact on procedural complications is yet to be determined.

Despite being a rare event, clinicians should have a high index of suspicion to hemothorax after thoracentesis. Vital signs instability, respiratory distress, hematocrit drop, and rapid pleural fluid reaccumulation within few hours after procedure should prompt further investigation [1].

The use of chest ultrasound has not been evaluated as a screening tool for postthoracentesis hemothorax. Promising results have been however reported in regard to ultrasound detection of traumatic hemothoraces showing high sensitivity and specificity [10]. Early fluid reaccumulation and complex echogenic or septated effusions on a postthoracentesis ultrasound can be proposed as early sonographic features to diagnose hemothorax [2, 11]. Compared to radiography, chest ultrasound has the advantages of easy portability, real time imaging, and absence of radiation. Add to that the fact that ultrasound has the ability to detect smaller volume of fluid and differentiate complex fluid from simple effusion [12]. These findings can be detected in the early hours after procedure leading to earlier intervention and management.

Our patient had multiple risk factors for bleeding complications including advanced renal insufficiency and history of coronary artery bypass surgery. Median sternotomy is believed to have produced anatomical variation of the patient's chest wall. Despite adhering to safety precautions and patient's tolerance to the procedure, ICA laceration led to accumulation of blood in the pleural space over a period of few hours. Initial pleural fluid analysis did not reveal any evidence of active bleeding. Upon clinical deterioration, bedside chest ultrasound provided an early suspicion of hemothorax after detection of fluid reaccumulation and transformation of anechoic simple fluid to complex septated effusion (Figure 5). Ideally procedural bleeding complications should be avoided through caution when dealing with high risk patients and possibly the utilization of vascular sonography. However, following ICA laceration, ultrasound findings can play a key role in prompt diagnosis resulting in early intervention and bleeding control.

## 4. Conclusion

Hemothorax is a rare fatal complication of thoracentesis. Population at risk can develop bleeding complications despite operator's adherence to proper technique. This case report emphasizes the role of bedside chest ultrasound as a simple screening tool for early detection of pleural fluid features suggestive of hemothorax. These findings should prompt further investigation and possible intervention to achieve hemostasis.
